# The Yeast Cell Fusion Protein Prm1p Requires Covalent Dimerization to Promote Membrane Fusion

**DOI:** 10.1371/journal.pone.0010593

**Published:** 2010-05-11

**Authors:** Alex Engel, Pablo S. Aguilar, Peter Walter

**Affiliations:** Howard Hughes Medical Institute and Department of Biochemistry and Biophysics, University of California San Francisco, San Francisco, California, United States of America; CNRS UMR6543, Université de Nice, Sophia Antipolis, France

## Abstract

Prm1p is a multipass membrane protein that promotes plasma membrane fusion during yeast mating. The mechanism by which Prm1p and other putative regulators of developmentally controlled cell-cell fusion events facilitate membrane fusion has remained largely elusive. Here, we report that Prm1p forms covalently linked homodimers. Covalent Prm1p dimer formation occurs via intermolecular disulfide bonds of two cysteines, Cys-120 and Cys-545. *PRM1* mutants in which these cysteines have been substituted are fusion defective. These *PRM1* mutants are normally expressed, retain homotypic interaction and can traffic to the fusion zone. Because *prm1-C120S* and *prm1-C545S* mutants can form covalent dimers when coexpressed with wild-type *PRM1*, an intermolecular C120-C545 disulfide linkage is inferred. Cys-120 is adjacent to a highly conserved hydrophobic domain. Mutation of a charged residue within this hydrophobic domain abrogates formation of covalent dimers, trafficking to the fusion zone, and fusion-promoting activity. The importance of intermolecular disulfide bonding informs models regarding the mechanism of Prm1-mediated cell-cell fusion.

## Introduction

Membrane fusion is a fundamental process that allows for the mixing of membrane bound compartments and their limiting membranes [1]. Eukaryotic cells use exquisitely regulated intracellular membrane fusion events to move membrane integral and soluble cargo through the various compartments of the secretory and endocytic pathways while maintaining compartmental identity [2]. Membrane fusion also occurs between cells, for example when gametes fuse their cell membranes to form zygotes and myoblasts fuse during the development of skeletal muscle [3], and for virus/cell fusion when enveloped viruses deliver their genome into host cells for infection [4]. Each of these processes requires a mechanism to fuse membranes in a specific, fast, and controlled manner.

The energy required to dehydrate and fuse lipid bilayers is too high to allow spontaneous membrane fusion to occur on a biologically reasonable time scale. Membrane fusion reactions are catalyzed by proteins collectively referred to as “fusases”. A protein family required for most intracellular fusion events, SNAREs (soluble *N*-ethylmaleimide-sensitive factor attachment protein receptors) use the energy of protein folding coupled to transmembrane anchors to pull membranes together and destabilize and fuse lipid bilayers [5]. Similarly, viral fusases refold from a metastable conformation to bring together a transmembrane anchor and a fusion peptide and achieve the same result [6], [7]. Though high-resolution structures of the soluble domains of SNARE complexes and both classes of viral fusases have been solved, the precise stoichiometry and geometry of the fusases in membranes during bilayer fusion is not known.

The fusases catalyze the formation of non-bilayer membrane states that can resolve into an aqueous pore connecting the two previously distinct compartments. The membrane fusion intermediates maintain the identity of the fusing compartments by preventing leakage of contents from the fusing compartments into the surrounding space. Both SNARE and viral fusion proceed through a hemifusion intermediate in which proximal leaflets of opposed membranes fuse to form a hemifusion stalk [8]. Current models predict that the expansion of the hemifusion stalk allows the distal leaflets to form a bilayer, and rupture of this bilayer results in a fusion pore, providing continuity between the fusing compartments and maintaining their identity. The proposed mechanism ascertains that no content leakage can occur and no membrane holes are formed. However, the fidelity of membrane fusion as catalyzed by SNAREs and viral fusases is not failsafe. Contents leakage has been observed in *in vitro* viral fusion systems [9], [10], and vacuoles containing high SNARE density lyse in a SNARE-dependent process [11]. The mechanism by which compartmental specificity is maintained *in vivo* may therefore be more complicated than previously appreciated, involving the ordered assembly of fusases and possibly additional fusase cofactors to maintain membrane and compartmental integrity [12].

Another membrane fusion event that exhibits loss of compartmental identity is cell-cell fusion during yeast mating. Haploid yeast cells of opposite mating type fuse to form a diploid cell—a gamete fusion event akin to sperm-egg fusion [3]. In the process of yeast mating, cells polarize towards mating partners using pheromone gradients, agglutinate, locally remove cell wall material to allow for membrane contact, and fuse cell membranes. A key regulator of the membrane fusion step of yeast cell fusion is Prm1p, a mating specific multipass membrane protein that localizes to the zone of cell fusion. Prm1p is only required in one partner of the mating pair to promote fusion; however, if both cells of the mating pair lack Prm1p, less than half of mating pairs successfully fuse [13]. Instead, two defective outcomes are observed: First, many mating pairs accumulate with membranes that remain unfused but are in very close apposition indicative of a failure to initiate bilayer fusion [13]. Second, many *prm1Δ*×*prm1Δ* mating pairs rupture [14]. The extent of mating pair lysis is Ca^2+^ dependent; in the absence of Ca^2+^ more than half of *prm1Δ*×*prm1Δ* mating pairs lyse, and, conversely, an increase in extracellular Ca^2+^ suppresses the lysis defect [15]. Mating pair lysis requires membrane contact [14], occurs with the same timing as membrane fusion [15], and is only observed in mutants of the cell membrane fusion step. Considering the membrane destabilizing activity of fusases, misregulation of the cell fusase is a plausible explanation of the observed aberrant cell lysis events.

**Figure 1:**
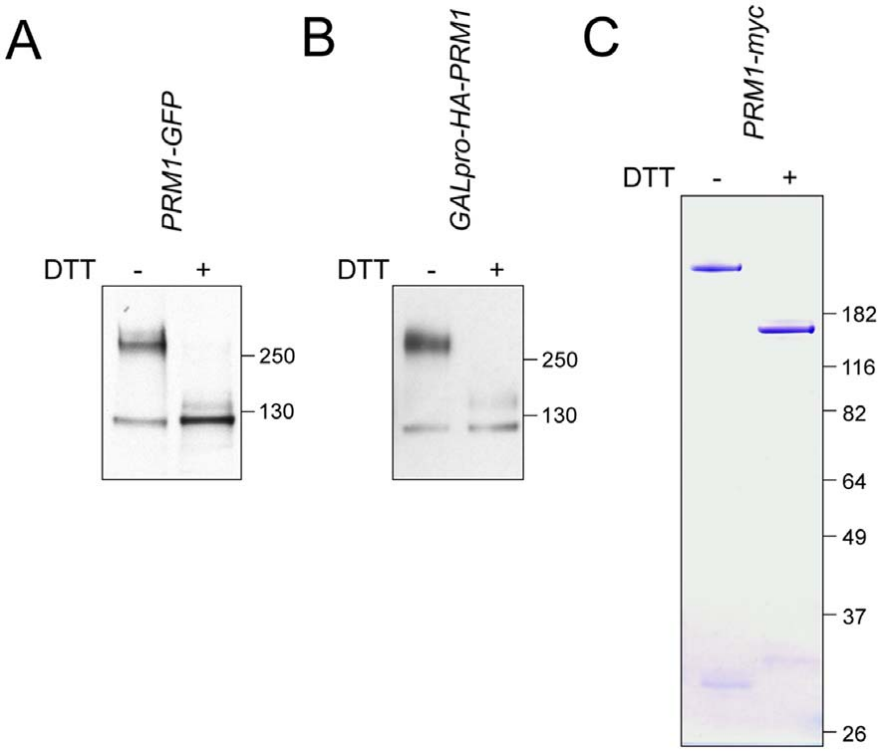
Prm1p is incorporated into a reduction-sensitive high molecular weight complex. (A) Anti-GFP Western blot on whole cell lysate of *PRM1-GFP MAT* **a** cells induced with 10 µg/ml α-factor for 90 min. (B) Anti-HA Western blot on whole cell lysate after galactose induction of *HA-PRM1*. (C) Prm1p was purified from a population of mating cells by α-myc immunoprecipitation, eluted with 2% SDS, run on a 10% bis-Tris polyacrylamide gel, and visualized by colloidal blue staining. Protein samples were reduced with 100 mM DTT.

A Prm1p homolog in the filamentous yeast *Neurospora crassa* similarly shows cell membrane fusion defects at both vegetative and sexual cell fusion steps [16]. In conjunction with Prm1p, another multipass membrane protein—Fig1p—is required for efficient membrane fusion [15]. Fig1p is a tetrapass membrane protein of the claudin-stargazing family [17]. A *Schizosaccharomyces pombe* Fig1p homolog Dni1p similarly promotes membrane fusion, in its absence membranes are juxtaposed without fusion and abnormal membrane structures are apparent [18]. Thus the machinery identified in S. cerevisiae appears conserved among other yeasts.

The mechanism by which Prm1p promotes membrane fusion over cell lysis is not well understood. In this study we demonstrate that Prm1p exists as a covalently linked homodimer. Furthermore, we show that the covalent linkage is necessary for Prm1p function, consistent with models in which Prm1p plays a role in confining the site of membrane fusion [12], [14], [15]. These results begin to describe the organization of the yeast cell fusion machinery and may be of general importance to other membrane fusion events.

## Results

### Prm1p forms a covalent homodimer

Proteins involved in fusion reactions often exist as multimers—enveloped virus fusases form homotrimers [6] and the developmentally important fusase EFF-1 involved in syncycium formation in *Caenorhabditis elegans* can oligomerize at the cell surface [19]. We noticed that the mobility of α-factor induced Prm1p in SDS-PAGE was remarkably slower when the protein sample was not reduced (Figure 1A). When reducing agent was omitted from the sample buffer, Prm1p migrated with an apparent mass greater than 250 kD. By contrast, if the protein samples were reduced with DTT prior to electrophoresis, Prm1p ran with an apparent molecular mass of 125 kD, consistent with the sum of the masses of monomeric Prm1p (73 kD), sugar modifications, and the GFP tag [13]. The mobility of unreduced Prm1p was not affected when cell lysis was carried out in the presence of iodoacetamide, establishing that covalent dimerization was not due to non-specific oxidation during sample preparation (data not shown).

The reduction-sensitive behavior of Prm1p in SDS-PAGE suggested that Prm1p exists as a covalent homo- or heterodimer. To see if formation of the high molecular weight complex required expression of additional mating specific genes or pheromone-induced MAPK signaling, we expressed Prm1p under the control of the *GAL1* promoter (Figure 1B). In this non-mating context, Prm1p was still found as part of a high molecular weight complex. This experiment also shows that dimer formation was not artifactually caused by the presence of the GFP moiety. Finally, we purified Prm1p from mating mixtures under native conditions to see if we could identify non-Prm1p interacting partners. The same reduction-sensitive behavior was observed with colloidal blue staining (Figure 1C). Notably, reduction of the Prm1p sample did reveal some faint, smaller molecular weight bands (∼30 kD), yet analysis of the unreduced sample by mass spectrometry identified only numerous Prm1p peptides without any significant coverage of any other protein (data not shown). Thus, the most plausible explanation is that the minor bands seen in the 30 kD region of the gel are non-specific proteolytic fragments of Prm1p. Taken together, these results suggest that the high molecular weight species is a covalently linked homodimer of Prm1p molecules.

**Figure 2:**
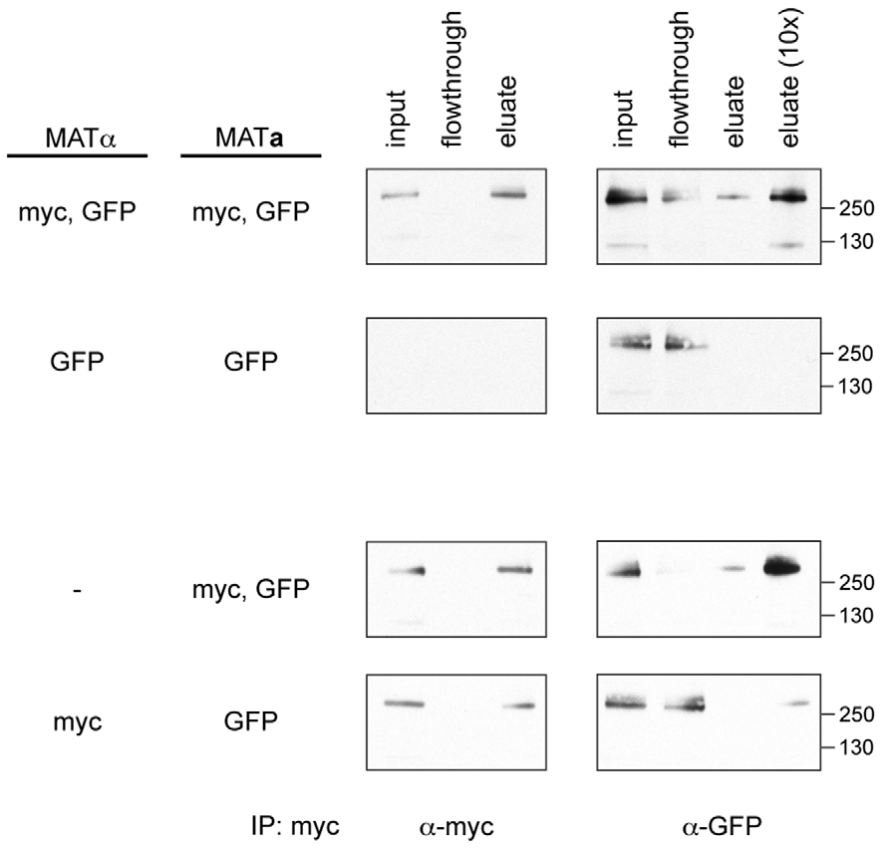
Prm1p forms covalent homodimers. *MAT* **a** and *MAT*α cells expressing epitope tagged *PRM1* as indicated were mated on YPD plates at 30°C for 3 h. After cell disruption, membrane proteins were solubilized in 1% Triton X-100 and immunoprecipitated using α-myc-Agarose. Western blotting with α-myc and α-GFP antibodies was used to assay pull-down efficiency and co-immunoprecipitation.

To test directly if Prm1p forms covalently linked homodimers, we coexpressed Prm1-myc and Prm1-GFP in both *MAT* **a** and α cells under the control of the *PRM1* promoter. These cells were mated on YPD to induce Prm1p expression in the natural mating context. Prm1-myc was immunoprecipiated, and co-precipitation of Prm1-GFP was monitored by Western blot (Figure 2, top row of panels). Prm1-myc was efficiently immunoprecipitated (Figure 2, left panels) and co-immunoprecipitation of Prm1-GFP with Prm1-myc was evident (Figure 2, top right panel). In the absence of Prm1-myc, we were unable to detect Prm1-GFP in the eluate of anti-myc immunoprecipitates (Figure 2, second row). As estimated by loading excess immunoprecipiation eluate, approximately 10% of Prm1-GFP co-eluted with Prm1-myc (Figure 2, top right panel). If both fusion proteins are expressed at similar levels, this 10% co-IP efficiency is lower than expected for an unbiased, obligate partnership, for which 50% of Prm1-GFP would be expected to co-precipitate. The reduced efficiency may represent a preference for self-associating with proteins translated from the same mRNA, which would deliver the monomeric building blocks in close spatial proximity.

Next, we asked if the Prm1p covalent dimers could interchange molecules by testing if new pairings could be formed after cell fusion. Prm1-myc was immunoprecipitated from mating mixtures in which approximately half of the input cells had formed zygotes. When the epitope-tagged *PRM1* alleles were co-expressed, Prm1-GFP co-immunoprecipitated with Prm1-myc (Figure 2, third row). However, when the expression of each *PRM1* allele was separated into opposite mating types, very little Prm1-GFP was apparent in the anti-myc eluate (Figure 2, bottom row). This small amount of associated Prm1-GFP likely represents dimerization of Prm1-myc and Prm1-GFP proteins synthesized *after* the zygote had formed. These results support a model in which Prm1p covalent dimerization occurs in the ER and no interchange occurs at the cell surface.

### Covalent dimerization is required for Prm1p activity

**Figure 3:**
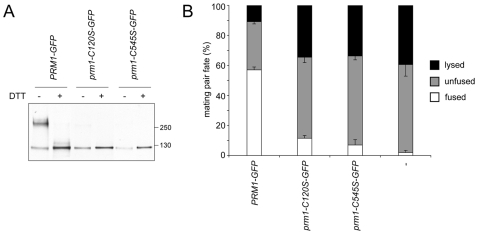
Two cysteines are required for formation of functional covalent Prm1p dimers. (A) Anti-GFP Western blot on whole cell lysate after expression of *PRM1-GFP* or cysteine-substituted mutants was induced with 10 µg/ml α-factor for 90 min. (B) *prm1Δ MAT*α cells expressing cytoplasmic GFP and *prm1Δ MAT* **a** cells bearing the indicated *PRM1* alleles on low copy plasmids were mated on EGTA-containing plates at 30°C for 3 h. Mating pairs were fixed and mating pair fate was scored microscopically by observing diffusion of cytoplasm throughout the mating pair (fusion) or loss of GFP signal and abnormal morphology (lysis).

Because the Prm1p covalent dimer was SDS-resistant and reduction-sensitive, we reasoned that the interaction between monomers is likely stabilized by intermolecular disulfide bridges. To test this possibility, we mutated each of the twelve Prm1p cysteines to serines and assayed the ability of these mutants to form covalent dimers. Of these twelve mutants, two (*prm1-C120S-GFP* and *prm1-C545S-GFP*) were unable to form covalent dimers while the rest were unaffected (Figure 3A and data not shown). As neither *prm1-C120S-GFP* nor *prm1-C545S-GFP* formed covalent dimers, these data suggest that Prm1p-Prm1p linkage occurs via two reciprocal intermolecular C120–C525 disulfide bonds. We tested the function and localization of the *prm1-C120S-GFP* and *prm1-C545S-GFP* alleles by expressing them in a *prm1Δ MAT* **a** background and mating to a *prm1Δ MAT*α strain. Fusion was scored microscopically by monitoring diffusion of cytoplasmic GFP expressed in the *MAT*α strain [13]. To enhance the penetrance of the *prm1Δ* phenotype, mating was performed on media supplemented with 20 mM EGTA [15] (Figure 3B, fourth bar). Under these conditions almost 60% of mating pairs successfully fused when the *prm1Δ* deletion was covered by *PRM1-GFP* (Figure 3B, first bar). By contrast, only 10% of mating pairs fused when the *PRM1* deletion was covered by *prm1-C120S-GFP*, and a similarly severe fusion defect was seen for *prm1-C545S-GFP* (Figure 3B, second and third bar). These data show that the cysteine mutation alleles retain some activity as *prm1Δ*×*prm1Δ* mating mixtures yield only 2% fused mating pairs. None of the other 10 cysteine-substituted alleles, which did not affect covalent dimerization, had reduced fusion activity relative to wild-type *PRM1-GFP* (data not shown). Because Cys-120 is adjacent to a highly hydrophobic series of amino acids, we also constructed substitutions with alanine and leucine to ensure that the introduction of the more polar serines was not interfering with the activity of this mutant. Both of these alleles behaved identically to *prm1-C120S-GFP* (data not shown). Though Cys-120 was initially predicted to reside within a putative transmembrane region, a recent report suggests that this hydrophobic stretch does not span the membrane [20].

**Figure 4:**
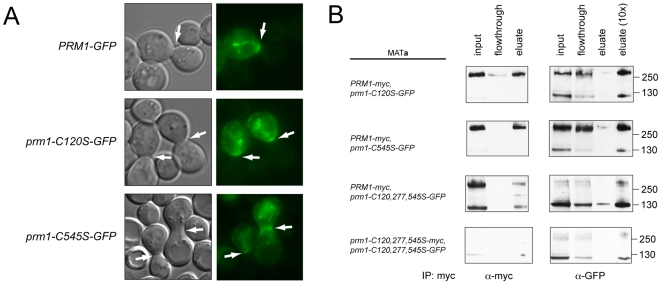
Prm1p localization and self-association are not affected by cysteine substitution. (A) Transmitted light and epifluorescence images of live mating pairs shortly after coupling. Prm1p and indicated alleles were C-terminally tagged with GFP. (B) *MAT* **a** cells expressing epitope tagged *PRM1* or mutant alleles as indicated were mated to wild-type *MAT*α cells on YPD plates at 30°C for 3 h. Myc-tagged fusion proteins were immunoprecipitated using α-myc-Agarose.

Despite the failure of *prm1-C120S-GFP* and *prm1-C545S-GFP* to support cell fusion, the protein products specified by these alleles were delivered to the site of cell-cell contact (Figure 4A, arrows). Thus, the fusion defect is not explained by a failure to deliver the cysteine-substituted proteins to the appropriate locale. A fraction of the prm1-C545S-GFP molecules may be incorrectly routed, possibly to the vacuole, as indicated by increased intracellular staining. Additionally, both prm1-C120S and prm1-C545S retained their ability to interact with wild-type Prm1p (Figure 4B). When both wild-type *PRM1-myc* and *prm1-C120S-GFP* or *prm1-C545S-GFP* were coexpressed from low copy plasmids, a significant fraction of the mutant proteins were integrated into covalent dimers (Figure 4B, right panels). These results confirm that intermolecular disulfide bonding occurs C120–C525. No bias was observed in interaction efficiency between covalently linked proteins compared to non-covalently associated proteins, demonstrating that these cysteines are not required for Prm1p to self-associate. By contrast, substituting all three cysteines originally predicted to reside in the lumenal/extracellular space (Cys-120, Cys-277 and Cys-545) prevented covalent dimerization with wild-type Prm1p (Figure 4B, third row), yet did not reduce co-immunoprecipitation efficiency. Interestingly, coexpression of *prm1-C120,277,545S-GFP*, but not *prm1-C120S-GFP* or *prm1-C545S-GFP*, prevented a fraction of wild-type Prm1p from forming a covalent dimer (Figure 4B, third row). We reason that the triply substituted mutant interacts with and sequesters wild-type Prm1p, but, unlike the singly substituted mutants, does not contain the cysteine(s) required to covalently bridge monomers.

### Charged residues adjacent to Cys-120 contribute to covalent dimer formation

**Figure 5:**
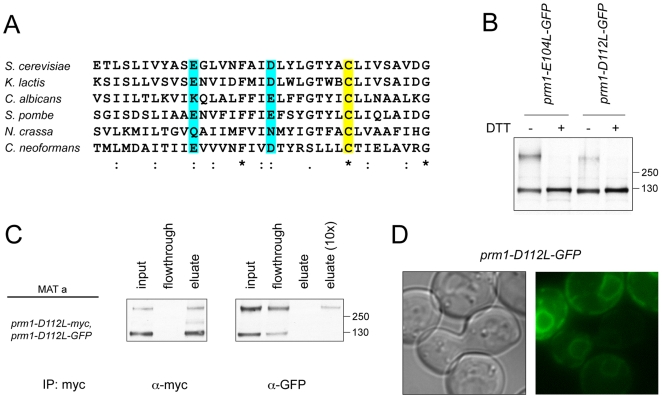
Charged residues within a conserved hydrophobic region adjacent to Cys-120 promote Prm1p covalent dimerization and polarized localization. (A) ClustalW alignment of the Prm1p hydrophobic region adjacent to Cys-120. (B) Anti-GFP Western blot on whole cell lysate after expression of *prm1-E104L-GFP* or *prm1-D112L-GFP* was induced with 10 µg/ml α-factor for 90 min. (C) *MAT* **a** cells coexpressing *prm1-D112L-GFP* and *prm1-D112L-myc* were mated to wild-type *MAT*α cells on YPD plates at 30°C for 3 h. Myc-tagged fusion proteins were immunoprecipitated using α-myc-Agarose. (D) Transmitted light and epifluorescence images of prm1D112LGFP in a live mating pair.

The two cysteines required for covalent dimerization are located within the most phylogenetically conserved stretches of Prm1p. Cys-120 is located adjacent to a highly conserved, largely hydrophobic sequence in the first extracellular loop [20]; Cys-545 is in the middle of the most conserved stretch of the second extracellular loop. The Cys-120-adjacent hydrophobic domain contains two charged residues (Glu-104 and Asp-112), which are well conserved among fungal homologs (Fig 5A). Due to their proximity to Cys-120, and given the evidence that Cys-120 forms an intermolecular disulfide linkage, we predicted that these residues would play important roles in the interaction between Prm1p molecules. Nonconservative substitution of Glu-104 with leucine only mildly compromised covalent dimer formation and fusion activity (Figure 5B and data not shown); by contrast, Asp-112 substitution with leucine significantly reduced covalent linkage (Fig 5B). As the *prm1-D112L-GFP* phenotype was more defective, we chose to characterize this mutant further. To test if prm1-D112L-GFP monomers do not interact as well as wild-type Prm1p monomers, we coexpressed *prm1-D112L-myc* and *prm1-D112L-GFP* and immunoprecipitated *prm1-D112L-myc* from mating cells (Fig 5C). We found that the majority of prm1-D112L-myc was not present as a covalent dimer (Fig. 5C, left panel, “input”). Prm1-D112L-GFP was more efficiently incorporated into covalent dimers when co-expressed with *prm1-D112L-myc*, presumably because the presence of both fusion protein variants of the mutant gene resulted in higher expression. Nonetheless, only a small fraction of prm1-D112L-GFP coprecipitated with prm1-D112L-myc (Fig. 5C, right panel “eluate” and “eluate 10x”), and this fraction was exclusively the covalently associated form. These results suggest that prm1-D112L mutants bind each other with reduced affinity.

We observed above that covalent dimerization was necessary for Prm1p activity based on the loss of fusion activity of the cysteine-substituted alleles. We thus expected that *prm1-D112L-GFP* would be similarly defective. Indeed, mating pairs of *prm1 MAT*α and *prm1 MAT* **a** cells expressing *prm1-D112L-GFP* exhibited a strong fusion defect (9% +/− 3% fused mating pairs, compared to 57% +/− 3% in wild-type controls). Additionally the prm1-D112L-GFP mutant failed to localize to the zone of cell fusion and was retained in the ER instead (Fig 5D). The mislocalization of prm1-D112L is consistent with the hypothesis that Prm1p dimerization, but not covalent linkage (see prm1-C120S-GFP and prm1-C545S-GFP, Fig. 4A), is a prerequisite for ER exit.

## Discussion

Prm1 plays an important role in the fusion of cell membranes during yeast mating. Without Prm1p, mating pairs arrest with membranes in close apposition or undergo cell lysis [13], [14]. It is not understood how Prm1p promotes membrane fusion and prevents cell lysis. In this study we show that Prm1p forms a covalent dimer, and that this covalent linkage is important for Prm1p function. While this manuscript was in preparation, Olmo & Grote [20] independently published a series of related experiments and reached the same conclusions.

There is strong evidence that Prm1p exists predominantly as a dimer covalently linked via intermolecular disulfide bridges: First, Prm1p migrates with an apparent mass of greater than 250 kD in the absence of reducing agent. This high molecular weight species is disassembled into Prm1p monomers by reducing agent. Second, Prm1p interacts with itself as shown by co-immunoprecipitation of differently tagged versions expressed in the same cell. Finally, the high molecular weight species does not form when Cys-120 or Cys-545 of Prm1p are mutated, implying reciprocal intermolecular C120–C545 disulfide bridges. Prm1p dimers probably form soon after protein synthesis and folding in the ER where they are stabilized by intermolecular disulfide bonds. Importantly, formation of covalently linked Prm1p dimers is necessary for the Prm1p fusion-promoting mechanism. Blocking intermolecular disulfide linkages by mutating relevant cysteines did not impinge upon Prm1p expression, block localization to the cell-cell fusion zone, or prevent self-association. The importance of the intermolecular C120–C545 linkage highlights a conserved hydrophobic domain directly N-terminal of Cys-120. Substitution of a charged residue within this hydrophobic domain, Asp-112, prevents Prm1p covalent dimerization and ER exit. This hydrophobic region is required for initial packaging of Prm1p into functional complexes, and it will be of great interest to examine if this region also has downstream roles at the membrane fusion step as suggested by its pronounced phylogenetic conservation (see below).

Prm1p covalent dimerization fits a model in which Prm1p plays a structural role in the membrane fusion machine [12], [14], [15]. In this model, Prm1p oligomers surround cell-cell fusase proteins and membrane fusion intermediates in a circular array. By forming such a structure, Prm1p may be able to influence membrane fusion by capturing fusase molecules in a cooperative arrangement. Covalent linkage of dimers would keep one interface of the Prm1p ring from dissociating, which could be necessary due to the high energies needed for membrane fusion and the vigorous conformational rearrangements accompanying the action of canonical fusases [21]. Another proposed function of this ring is to limit the expansion of membrane holes by corralling lipids within a narrow zone defining a membrane fusion microenvironment. Such holes could be an off-pathway outcome of membrane fusion, or, as predicted by molecular simulations, a true intermediate [22], and we have speculated before that they may arise as a direct consequences of fusase activation [12], [15]. The intermolecular disulfide bridges could increase the effectiveness of a Prm1p barrier to lipid diffusion, especially considering the hydrophobic environment in which Cys-120 is located.

Such functions have been either hypothesized or demonstrated for other classes of membrane fusion. In the case of SNARE-mediated membrane fusion, the prevailing model is that multiple trans-SNARE pairs assemble into a ring-like structure. This structure has been observed in reconstituted SNARE protein membrane docking by atomic force microscopy [23]. Unknown factors have been hypothesized to organize the fusases in this configuration, as this complex is unlikely to form spontaneously [24]. In support of the Prm1p-corrall model, the restriction of lipid flow by the assembled fusion machine has been observed during HA-catalyzed membrane fusion [25]. A ring-shaped oligomer consisting of many HA trimers is thought to surround the hemifusion stalk and prevent diffusion of lipids between merged *cis* leaflets. A few predictions can be made if Prm1p indeed can organize fusase complexes or prevent lipid diffusion during the membrane fusion process. First, Prm1p would need to physically interact with the fusion machine. Thus, Prm1p remains a promising handle for biochemical or genetic identification of the yeast cell fusase. Second, Prm1p covalent dimers should be able to form higher order oligomers, either by themselves or in concert with other proteins of the cell fusion machinery.

It is also possible that Prm1p can directly act as a fusase alone or in conjunction with other proteins. If this were the case, the intermolecular disulfide bonds may serve to lock Prm1p in a metastable state, akin to HA in its neutral pH conformation. Conformational rearrangements from this metastable state upon Prm1p activation would provide the energy for membrane fusion. An alternative and not mutually exclusive function of the putative C120–C545 intermolecular linkage could be to control insertion of the hydrophobic region N-terminal of Cys-120 either in same bilayer in which Prm1p is anchored or in the closely juxtaposed cell membrane of the mating partner. This could destabilize the targeted bilayer and promote initiation of bilayer mixing. Though the intermolecular disulfide linkage is clearly required for fusion-competence of Prm1p, the breaking of this bond (conceivably achieved by the release of a burst of reductants at the site of fusion) may control membrane partitioning of the hydrophobic region analogous to exposure and positioning of the fusion peptide of HA that is liberated by conformation rearrangement upon exposure to acidic pH.

## Materials and Methods

### Media and Yeast Strains

Synthetic complete (SC), and complex (YPD) media were prepared and supplemented with 2% glucose using reagents from Difco Inc. and Sigma Chemical Company.

All strains used in this study are derivatives of wild-type strain W303, the *prm1Δ* mutant strains were generated in a previous study [13], and the genomic fusion of *GAL_PRO_-HA-PRM1* was generated by PCR transformation technique [26].

### Plasmid construction

*PRM1-GFP*, including 507 bp of the 5′ promoter region and the *ADH1* terminator 3′ of the GFP sequence, was amplified from genomic DNA of a *PRM1-GFP* strain and cloned into pRS315 by gap repair. Cysteine substitution was achieved by site directed mutagenesis. Myc-tagged *PRM1* constructs were generated by gap repair of the corresponding *PRM1-GFP* plasmid digested with AscI/PacI using PCR product amplified from genomic DNA of a *PRM1-myc* strain. *PRM1* alleles were subcloned into pRS314 as a SacI/XhoI fragment. Plasmids constructed for this study are listed in Table 1.

**Table 1:** Plasmids constructed for this study.

Plasmid name	*PRM1* allele	Parental plasmid
pAE18	*PRM1GFP*	pRS315
pAE28	*prm1-C6S-GFP*	pRS315
pAE29	*prm1-C55S-GFP*	pRS315
pAE20	*prm1-C120S-GFP*	pRS315
pAE21	*prm1-C277S-GFP*	pRS315
pAE30	*prm1-C302S-GFP*	pRS315
pAE31	*prm1-C308S-GFP*	pRS315
pAE32	*prm1-C329S-GFP*	pRS315
pAE33	*prm1-C377S-GFP*	pRS315
pAE34	*prm1-C424S-GFP*	pRS315
pAE35	*prm1-C436S-GFP*	pRS315
pAE36	*prm1-C438S-GFP*	pRS315
pAE22	*prm1-C545S-GFP*	pRS315
pAE37	*prm1-C120,277,545S-GFP*	pRS315
pAE38	*prm1-C120,277,545S-myc*	pRS314
pAE42	*prm1-E104L-GFP*	pRS315
pAE27	*prm1-D112L-GFP*	pRS315
pAE43	*prm1-D112L-myc*	pRS314

### Immunoprecipitations

50 OD units of each mating type were filtered onto 85 mm 0.45 µm HATF membranes (Millipore) and incubated for 3 h at 30°C. Cells were collected from the filters by vortexing in 10 ml YPD and pelleted at low speeds in an IEC clinical centrifuge. Pellets were resuspended in IP buffer (50 mM HEPES, 100 mM KOAc, 2 mM Mg(OAc)_2_, 1 mM PMSF, 1 mM EDTA, supplemented with the Complete protease inhibitor (Roche)), and cells were disrupted by bead beating with 0.5 mm glass beads (BioSpec Products, Inc.) for a total of 5 min in one min intervals alternating with ice incubations. After unlysed cells and large debris were removed by a 1000 RPM microcentrifugation step, the cell lysate was spun at 20K×g for 20 min. The membrane pellet was resuspended in IP buffer + 1% Triton X-100 (Pierce) for 2 h rotating at 4°C. Unsolublized membrane was pelleted in another 20K×g centrifugation step. The supernatant was applied to 30 µl of equilibrated agarose-coupled 9E10 anti-c-myc antibody slurry (Santa Cruz Biotechnology) and rotated for 2 h at 4°C. The beads were washed with 25 ml IP buffer + 1% Triton X-100 and bound proteins were eluted in after shaking at 50°C in PBS with 2% SDS for 5 min.

### Quantitative cell fusion assay

Mating pair fate was scored microscopically as previously described [13].

### Microscopy

Fluorescence and DIC microscopy was performed using an Axiovert 200 M microscope (Zeiss), equipped with an X-cite 120 mercury arc lamp (EXFO), and an Orca ER camera (Hamamatsu). Metamorph was used for data collection.
